# Overall survival and prognostic factors in young women with breast cancer: a retrospective cohort study from Southern Thailand

**DOI:** 10.1186/s12957-026-04349-9

**Published:** 2026-04-15

**Authors:** Phungern Khongthong, Tanaporn Prateepchaiboon, Tanyaporn Ruangsuwan

**Affiliations:** Hatyai Hospital, Songkhla, Thailand

**Keywords:** Breast cancer, Young women, Overall survival, Prognostic factors, Cox proportional hazards model, Thailand

## Abstract

**Background:**

Breast cancer diagnosed at a young age is often associated with aggressive tumor characteristics and poorer survival outcomes. In Southeast Asia, data on overall survival and prognostic factors among young women with breast cancer remain limited, particularly from real-world clinical settings with complete mortality ascertainment. This study aimed to describe survival outcomes and identify independent prognostic factors among women aged 45 years or younger with breast cancer at a major provincial referral hospital in Southern Thailand.

**Methods:**

We conducted a retrospective cohort study of women aged 45 years or younger diagnosed with histologically confirmed breast cancer at Hat Yai Hospital, Southern Thailand, between 2013 and 2018. Mortality was ascertained through linkage with the Thai National Civil Registration database. Molecular subtypes were classified using immunohistochemistry surrogate criteria per the St. Gallen 2013 consensus. Missing data were handled using multiple imputation by chained equations (MICE, m = 10). Overall survival (OS), disease-free survival (DFS), and distant disease-free survival (DDFS) were estimated using the Kaplan–Meier method. Prognostic factors were evaluated using univariable and multivariable Cox proportional hazards regression with the revised model including disease stage, tumour grade, molecular subtype, surgery, and hormone therapy.

**Results:**

A total of 293 young women were included. Median follow-up was 93.3 months (95% CI: 89.8–98.2). The 3-year and 5-year OS rates were 75.8% (95% CI: 71.0–80.8%) and 66.2% (95% CI: 61.0–71.9%), respectively. Five-year OS by stage ranged from 87.1% (Stage I) to 33.3% (Stage IV). The 5-year DFS and DDFS were 58.0% and 61.8%, respectively. The most common molecular subtype was HR+/HER2- (52.0%), followed by HR-/HER2- (22.3%), HR+/HER2+ (16.0%), and HR-/HER2+ (9.7%). In multivariable analysis, HR+/HER2 + subtype was independently associated with poorer overall survival compared with HR+/HER2- (adjusted HR = 2.08, 95% CI: 1.07–4.07, *p* = 0.032). Hormone therapy receipt was independently associated with better overall survival (adjusted HR = 0.20, 95% CI: 0.10–0.42, *p* < 0.001). Receipt of surgery was strongly associated with overall survival but largely reflected disease operability rather than an independent treatment effect.

**Conclusions:**

This study provides systematically ascertained, registry-linked survival benchmarks for young-onset breast cancer in Southern Thailand. HR+/HER2 + subtype and hormone therapy receipt were independent prognostic factors. The relatively lower stage-specific survival rates compared with high-income country benchmarks highlight the need for improved access to HER2-targeted therapy and endocrine therapy in this resource-limited setting.

**Supplementary Information:**

The online version contains supplementary material available at 10.1186/s12957-026-04349-9.

## Background

Breast cancer is the most commonly diagnosed cancer in women globally and remains a leading cause of cancer-related mortality worldwide [1]. Despite rising incidence with age, a significant proportion of breast cancer diagnoses in low- and middle-income countries affect younger women, reflecting both biological and epidemiological differences from high-income country patterns [2, 3]. Breast cancer diagnosed at a young age — commonly defined as 45 years or younger — is frequently associated with more aggressive tumour biology, advanced stage at presentation, and inferior survival outcomes compared with older women, making young-onset breast cancer a distinct clinical and public health challenge [4, 5].

Young women with breast cancer commonly present with unfavourable clinicopathological characteristics, including higher tumour grade, hormone receptor-negative disease, and increased rates of HER2 overexpression [6]. The molecular subtype distribution in young women shows a higher prevalence of HR-/HER2- and HER2-positive breast cancers, both of which are associated with poorer prognosis [7, 8]. Asian populations, including Thai women, this biological shift toward more aggressive subtypes at younger ages is well-documented, with peak breast cancer incidence occurring approximately one to two decades earlier than in Western populations [9, 10]. The absence of routine mammographic screening in younger age groups may further contribute to delayed diagnosis and more advanced disease at presentation [11].

Evidence regarding the independent prognostic impact of young age has been inconsistent. Earlier studies suggested that poorer survival in younger patients may be largely attributable to advanced stage and aggressive tumor characteristics rather than age itself [12, 13]. More recent meta-analyses and large cohort studies have demonstrated that young age — particularly below 40 years — is independently associated with worse disease-free and overall survival even after adjustment for tumour biology and treatment-related factors [14, 15].

In Southeast Asia, where breast cancer among younger women is relatively common, data on overall survival and prognostic factors remain limited, particularly from real-world clinical settings [16, 17]. Existing studies are frequently constrained by small sample sizes, incomplete follow-up, or reliance on institutional mortality records that do not capture deaths occurring outside the treating hospital [18]. In Thailand — particularly in Southern regions, where a large provincial referral hospital serves a geographically dispersed population of predominantly Thai ethnicity — comprehensive, long-term survival data for young women with breast cancer are scarce. Access to HER2-targeted therapy and genetic risk assessment was limited during the study period, reflecting resource constraints common to provincial Southeast Asian healthcare settings, and the real-world survival outcomes achievable in this context are not well characterised.

Accurate mortality ascertainment in retrospective cohort studies requires linkage beyond institutional medical records. Linkage with national civil registration systems provides a robust and cost-effective approach to capturing deaths regardless of whether patients remained in active clinical follow-up, substantially reducing the informative censoring that commonly biases hospital-based survival analyses. This methodological strength distinguishes our study from many existing Southeast Asian breast cancer series.

We therefore conducted a retrospective cohort study of women aged 45 years or younger diagnosed with breast cancer at Hat Yai Hospital, Southern Thailand, between 2013 and 2018. The primary objective was to estimate overall survival, disease-free survival, and distant disease-free survival, and to identify independent prognostic factors associated with mortality using Kaplan–Meier methods and multivariable Cox proportional hazards regression. Molecular subtypes were classified using immunohistochemistry surrogate criteria to provide clinically meaningful prognostic groupings. The study provides the first systematically ascertained, registry-linked survival benchmarks for this population, with direct applicability to prognostic counselling and treatment planning at comparable provincial Southeast Asian centres.

## Methods

### Study design and population

This retrospective cohort study included women aged 45 years or younger who were diagnosed with histologically confirmed breast cancer at Hatyai Hospital, Southern Thailand, between January 2013 and December 2018. Hatyai Hospital is the major tertiary referral centre for Southern Thailand, serving a geographically dispersed population of predominantly Thai ethnicity, including both Buddhist Thai and Muslim Thai communities. Patients were identified from institutional medical records. All patients were of Thai ethnicity; ethnicity was not further subdivided in data collection. Survival status was ascertained through linkage with the Thai National Civil Registration database, which records all deaths regardless of cause or location, ensuring near-complete mortality ascertainment independent of whether patients remained in active clinical follow-up at our institution. Patient selection and application of eligibility criteria are summarised in Supplementary Figure S1.

### Data collection and variables

Demographic, clinicopathological, and treatment-related variables were extracted from institutional medical records, including age at diagnosis, body mass index (BMI), disease stage, histological type, tumour grade, hormone receptor status (oestrogen receptor and progesterone receptor), HER2 status, type of surgery, neoadjuvant chemotherapy, adjuvant chemotherapy, adjuvant radiotherapy, and hormone therapy. Trastuzumab receipt was recorded separately; adjuvant trastuzumab became available at our institution after 2015 and was reimbursed exclusively for patients covered under the Civil Servant Medical Benefit Scheme with lymph node-positive, HER2-positive disease, in accordance with Thai national reimbursement policy during the study period.

Disease staging was performed according to the American Joint Committee on Cancer (AJCC) staging system based on clinical and pathological assessment. Pre-operative imaging included chest X-ray and upper abdominal ultrasound for all patients. CT imaging of the chest and abdomen was performed selectively for patients with clinically node-positive disease or primary tumour size greater than T3 (> 5 cm), in accordance with institutional protocols and available imaging resources. PET-CT scanning was not routinely performed, as this modality was not reimbursed under any Thai public health insurance scheme and was not available at our institution during the study period. In patients for whom Stage III disease was identified post-operatively based on pathological findings, CT staging was completed after surgery where clinically indicated.

Molecular subtypes were classified using immunohistochemistry (IHC) surrogate criteria according to the St. Gallen International Expert Consensus (2013) as follows: HR+/HER2- (ER-positive and/or PR-positive, HER2-negative); HR+/HER2+ (ER-positive and/or PR-positive, HER2-positive); HR-/HER2+ (ER-negative, PR-negative, HER2-positive); and HR-/HER2- breast cancer (Triple Negative) (Triple Negative; ER-negative, PR-negative, HER2-negative). HER2 IHC 2+ (equivocal) results were classified as HER2-negative in the absence of fluorescence in situ hybridisation (FISH) confirmation, as FISH testing was unavailable for 92.5% of equivocal cases. HR+/HER2- was designated as the reference category.

The extent of missing data is summarised in Table 1. Missing data were present in disease stage (*n* = 36, 12.3%), tumour grade (*n* = 39, 13.3%), HER2 status (*n* = 24, 8.2%), and hormone therapy (*n* = 107, 36.5%). Missingness rates differed by survival status — disease stage was missing in 5.6% of survivors versus 22.8% of deceased patients, and hormone therapy was missing in 31.3% of survivors versus 44.7% of deceased patients — indicating that data were not missing completely at random. Formal assessment confirmed that hormone therapy missingness was structurally associated with molecular subtype (χ²=259.99, *p* < 0.001), reflecting the absence of hormone therapy records in receptor-negative patients who do not receive endocrine therapy. Missing disease stage was not modelled as a separate covariate category in regression analyses, as this would not represent a clinically or biologically meaningful entity.

**Table 1:** Baseline clinicopathological characteristics by survival status

Characteristic	Overall (*N* = 293)	Alive (*N* = 179)	Dead (*N* = 114)	*p*-value
Age (years), median (IQR)	40.0 (37.0–43.0)	41.0 (37.0–43.0)	40.0 (35.2–43.0)	0.657
BMI (kg/m²), median (IQR)	23.0 (20.3–26.9)	23.1 (20.8–26.9)	22.7 (19.6–26.9)	0.367
Disease stage, n (%) [missing = 36 (12.3%)]				
Stage I	31 (12.1)	26 (15.4)	5 (5.7)	**< 0.001**
Stage II	124 (48.2)	95 (56.2)	29 (33.0)	
Stage III	78 (30.4)	41 (24.3)	37 (42.0)	
Stage IV	24 (9.3)	7 (4.1)	17 (19.3)	
Tumour grade, n (%) [missing = 39 (13.3%)]				
Grade I	36 (14.2)	31 (19.9)	5 (5.1)	**0.002**
Grade II	120 (47.2)	73 (46.8)	47 (48.0)	
Grade III	98 (38.6)	52 (33.3)	46 (46.9)	
Oestrogen receptor, n (%) [missing = 16 (5.5%)]				
Positive	186 (65.5)	124 (69.3)	62 (54.4)	**0.011**
Negative	91 (32.0)	44 (24.6)	47 (41.2)	
Progesterone receptor, n (%) [missing = 16 (5.5%)]				
Positive	159 (56.0)	107 (59.8)	52 (45.6)	**0.025**
Negative	118 (41.5)	61 (34.1)	57 (50.0)	
HER2 status, n (%) [missing = 24 (8.2%)]				
Positive	73 (27.7)	36 (20.1)	37 (32.5)	0.058
Equivocal	65 (24.6)	42 (23.5)	23 (20.2)	
Negative	155 (58.7)	101 (56.4)	54 (47.4)	
Molecular subtype, n (%) [missing = 24 (8.2%)]				
HR+/HER2-	140 (52.0)	91 (50.8)	49 (43.0)	**< 0.001**
HR+/HER2+	43 (16.0)	20 (11.2)	23 (20.2)	
HR-/HER2+	26 (9.7)	13 (7.3)	13 (11.4)	
HR-/HER2-	60 (22.3)	45 (25.1)	15 (13.2)	
Surgery, n (%)				
Yes	256 (87.4)	174 (97.2)	82 (71.9)	**< 0.001**
No	37 (12.6)	5 (2.8)	32 (28.1)	
Chemotherapy, n (%) [missing = 1 (0.3%)]				
Yes	251 (86.0)	138 (77.1)	113 (99.1)	**< 0.001**
No	41 (14.0)	41 (22.9)	0 (0.0)	
Hormone therapy, n (%) [missing = 107 (36.5%)]				
Yes	122 (65.6)	110 (61.5)	12 (10.5)	**< 0.001**
No	64 (34.4)	13 (7.3)	51 (44.7)	

Values are median (IQR) for continuous variables and n (%) for categorical variables

*BMI* = body mass index, *HER2* human epidermal growth factor receptor 2, *HR-/HER2 * Triple Negative Breast Cancer

Bold *p*-values indicate *p* < 0.05. IQR = interquartile range

*p*-values: Mann-Whitney U test for continuous variables; chi-square test for categorical variables

### Outcome definition

The primary outcome was overall survival (OS), defined as the time from the date of histologically confirmed breast cancer diagnosis to death from any cause. Overall survival was ascertained through linkage with the Thai National Civil Registration database, which records all deaths regardless of cause or location. As cause-of-death data were not consistently available in this retrospective cohort, all deaths were treated as events regardless of cause. Given the young age of this cohort (≤ 45 years), competing mortality from non-breast cancer causes is expected to be minimal, and overall survival is considered a close approximation of breast cancer-specific survival in this population. Patients who were alive at the end of follow-up were censored on the date of last linkage with the civil registration database.

Secondary outcomes were disease-free survival (DFS) and distant disease-free survival (DDFS). DFS was defined as the time from diagnosis to first recurrence at any site (locoregional or distant) or death from any cause, whichever occurred first. DDFS was defined as the time from diagnosis to first distant recurrence (visceral, bone, or contralateral disease) or death from any cause, whichever occurred first. Site of recurrence was classified as locoregional (local breast or regional lymph node recurrence), distant, or both. Patients without a recurrence event who were alive at the end of follow-up were censored at the date of last contact.

### Statistical analysis

Descriptive statistics were used to summarise baseline characteristics. Continuous variables were reported as median with interquartile range (IQR) and compared between groups using the Mann-Whitney U test. Categorical variables were summarised as frequencies and percentages and compared using the chi-square test or Fisher’s exact test, as appropriate. Median follow-up time was calculated using the reverse Kaplan–Meier method applied to censored observations.

Overall survival (OS), disease-free survival (DFS), and distant disease-free survival (DDFS) were estimated using the Kaplan–Meier method and compared across groups using the log-rank test. Survival rates at 3 and 5 years were extracted from Kaplan–Meier estimates with Greenwood standard errors.

#### Missing data

 Missing data were present in disease stage (*n* = 36, 12.3%), tumour grade (*n* = 39, 13.3%), HER2 status (*n* = 24, 8.2%), and hormone therapy (*n* = 107, 36.5%). The missingness mechanism was formally assessed using chi-square tests associating missingness indicators with observed clinical variables. Disease stage missingness was significantly associated with survival status (χ²=17.60, df = 1, *p* < 0.001), and hormone therapy missingness was structurally associated with molecular subtype (χ²=259.99, df = 3, *p* < 0.001), reflecting the absence of hormone therapy records in receptor-negative patients who do not receive endocrine therapy. HER2 missingness was associated with disease stage (χ²=11.32, df = 3, *p* = 0.010). These findings confirmed that data were not missing completely at random (MCAR). A missing at random (MAR) assumption conditional on observed covariates was considered most plausible; however, missing not at random (MNAR) cannot be entirely excluded for disease stage and hormone therapy, and this is acknowledged as a limitation.

Missing data were handled using multiple imputation by chained equations (MICE), implemented in the *mice* package in R (version 3.16.0). Ten imputed datasets (m = 10) were generated using predictive mean matching for continuous variables and logistic regression for binary variables. Auxiliary variables included age, BMI, surgery, chemotherapy, survival status, and follow-up time. Convergence was confirmed by visual inspection of trace plots. Pooled estimates were derived using Rubin’s Rules. Missing disease stage was not modelled as a separate covariate category in regression analyses, as this would not represent a clinically or biologically meaningful entity.

#### Univariable and multivariable Cox regression

 Prognostic factors for overall survival were evaluated using univariable and multivariable Cox proportional hazards regression. For the multivariable model, variable selection was guided by clinical relevance and statistical considerations regarding model stability. The final multivariable model included five covariates: disease stage, tumour grade, molecular subtype, surgery, and hormone therapy. With 114 events and 10 model parameters after categorical expansion, the events-per-variable (EPV) ratio was 11.4, meeting the recommended minimum threshold of 10 for stable Cox regression estimates. To address collinearity between hormone receptor variables, molecular subtype was used as a composite variable replacing separate ER, PR, and HER2 covariates. Chemotherapy was excluded from the multivariable model as it was not significant in univariable analysis (HR = 1.54, 95% CI: 0.94–2.53, *p* = 0.085) and 86.0% of patients received chemotherapy, precluding meaningful adjustment. Age and BMI were excluded as neither was significantly associated with survival in univariable analysis. Multicollinearity in the revised model was assessed using the generalised variance inflation factor (GVIF) implemented in the *car* package in R; all GVIF^(1/(2×Df)) values were below 2.0 (range: 1.07–1.43), confirming the absence of problematic multicollinearity. Results were reported as hazard ratios (HR) or adjusted hazard ratios (aHR) with 95% confidence intervals (CIs). As this was a retrospective observational cohort, all associations between treatment variables and survival should be interpreted as prognostic associations rather than causal treatment effects.

#### Proportional hazards assumption

 The proportional hazards assumption was formally evaluated using three complementary approaches: scaled Schoenfeld residuals (*cox.zph* function in R), graphical inspection of complementary log-log (cloglog) survival curves, and consideration of time-dependent covariate extensions. No statistically significant violation was detected for any individual covariate or globally (global Schoenfeld test: χ²=6.50, df = 9, *p* = 0.69). Complementary log-log survival curves demonstrated approximately parallel lines across all covariate categories (Supplementary Figure S2). For surgery, mild attenuation of the gap between groups was observed at later timepoints; however, the Schoenfeld residual test confirmed no statistically significant violation (*p* = 0.58), and this pattern is clinically consistent with the known early mortality benefit of surgical resection. Time-dependent covariate modelling was not required.

#### Sensitivity analyses

 Three pre-specified sensitivity analyses were performed. First, a complete case analysis restricted to patients with no missing values across all model covariates was conducted (Supplementary Table S1). Second, the multivariable model was restricted to patients with Stage I–III disease, excluding Stage IV patients, to avoid conflating the prognostic role of surgical treatment with the inherent survival disadvantage of metastatic disease (Supplementary Table S2). Third, a delta-adjusted pattern mixture sensitivity analysis was performed for hormone therapy to assess robustness to the MNAR assumption, by systematically shifting imputed hormone therapy values across five log-odds perturbation scenarios (δ = −1, − 0.5, 0, + 0.5, + 1). A two-sided p-value of less than 0.05 was considered statistically significant throughout.

All statistical analyses were conducted using R version 4.5.1 (R Foundation for Statistical Computing, Vienna, Austria) within the RStudio integrated development environment (RStudio PBC, Boston, MA, USA). Key packages used included *survival* (Cox regression and Kaplan–Meier analysis), *mice* (multiple imputation), *car* (GVIF assessment), *gtsummary* (table generation), and *survminer* (Kaplan–Meier visualisation). An artificial intelligence-based language model (Claude, Anthropic) was used to assist with language editing, statistical code, and formatting. The authors reviewed, edited, and take full responsibility for the content and conclusions of the manuscript.

### Ethics approval

This study was conducted in accordance with the Declaration of Helsinki. Ethical approval was obtained from the Human Research Ethics Committee of Hatyai Hospital (approval number HYH EC 110-68-01). Due to the retrospective nature of the study and use of de-identified data, the requirement for informed consent was waived.

## Results

### Patient characteristics

A total of 293 young women with breast cancer were included in the analysis. Of these, 179 patients (61.1%) were alive and 114 patients (38.9%) had died by the end of follow-up. The median follow-up time was 93.3 months (95% CI: 89.8–98.2 months), calculated using the reverse Kaplan–Meier method. The clinicopathological characteristics of the study population stratified by survival status are summarised in Table 1.

The median age at diagnosis was similar between survivors and non-survivors (41.0 years [IQR: 37.0–43.0] vs. 40.0 years [IQR: 35.3–43.0], *p* = 0.657). Median BMI also did not differ significantly between groups (23.1 kg/m² [IQR: 20.8–26.9] vs. 22.7 kg/m² [IQR: 19.6–26.9], *p* = 0.367).

Disease stage at diagnosis differed markedly by survival status (*p* < 0.001). Survivors were more likely to present with early-stage disease (Stage I–II: 67.6% vs. 29.8%), whereas advanced-stage disease was more frequent among non-survivors (Stage III–IV: 47.4% vs. 26.8%). Missing stage data were substantially more common in deceased patients (22.8%) than in survivors (5.6%), consistent with patients who died early before staging was completed. Tumour grade was significantly associated with survival status (*p* = 0.002), with a higher proportion of poorly differentiated (Grade III) tumours among non-survivors (46.9% vs. 33.3%). Histological subtype did not differ significantly between groups (*p* = 0.758).

With respect to molecular subtype, HR+/HER2- was the most common subtype (*n* = 140, 52.0% of classifiable patients), followed by HR-/HER2- (*n* = 60, 22.3%), HR+/HER2+ (*n* = 43, 16.0%), and HR-/HER2+ (*n* = 26, 9.7%). Twenty-four patients (8.2%) were unclassifiable due to missing receptor data. Oestrogen receptor-positive tumours were more common among survivors (69.3% vs. 54.4%, *p* = 0.011), as were progesterone receptor-positive tumours (59.8% vs. 45.6%, *p* = 0.025). HER2 status showed a trend toward association with survival but did not reach statistical significance (*p* = 0.058).

Treatment patterns differed substantially by survival status. Patients who survived were significantly more likely to have undergone surgery (97.2% vs. 66.7%), received chemotherapy (77.1% vs. 46.5%), and received hormone therapy (61.5% vs. 34.2%; all *p* < 0.001), as shown in Table 1. Of 292 patients with chemotherapy data, 52 (17.8%) received neoadjuvant chemotherapy — predominantly in Stage III patients (28/52, 53.8%) — and 199 (67.9%) received adjuvant chemotherapy. Adjuvant trastuzumab was received by 12 patients (4.1%), reflecting the restricted insurance reimbursement applicable during the study period.

### Survival outcomes

The 3-year and 5-year overall survival rates for the entire cohort were 75.8% (95% CI: 71.0–80.8%) and 66.2% (95% CI: 61.0–71.9%), respectively. Median overall survival was not reached for the entire cohort.

**Fig. 1:**
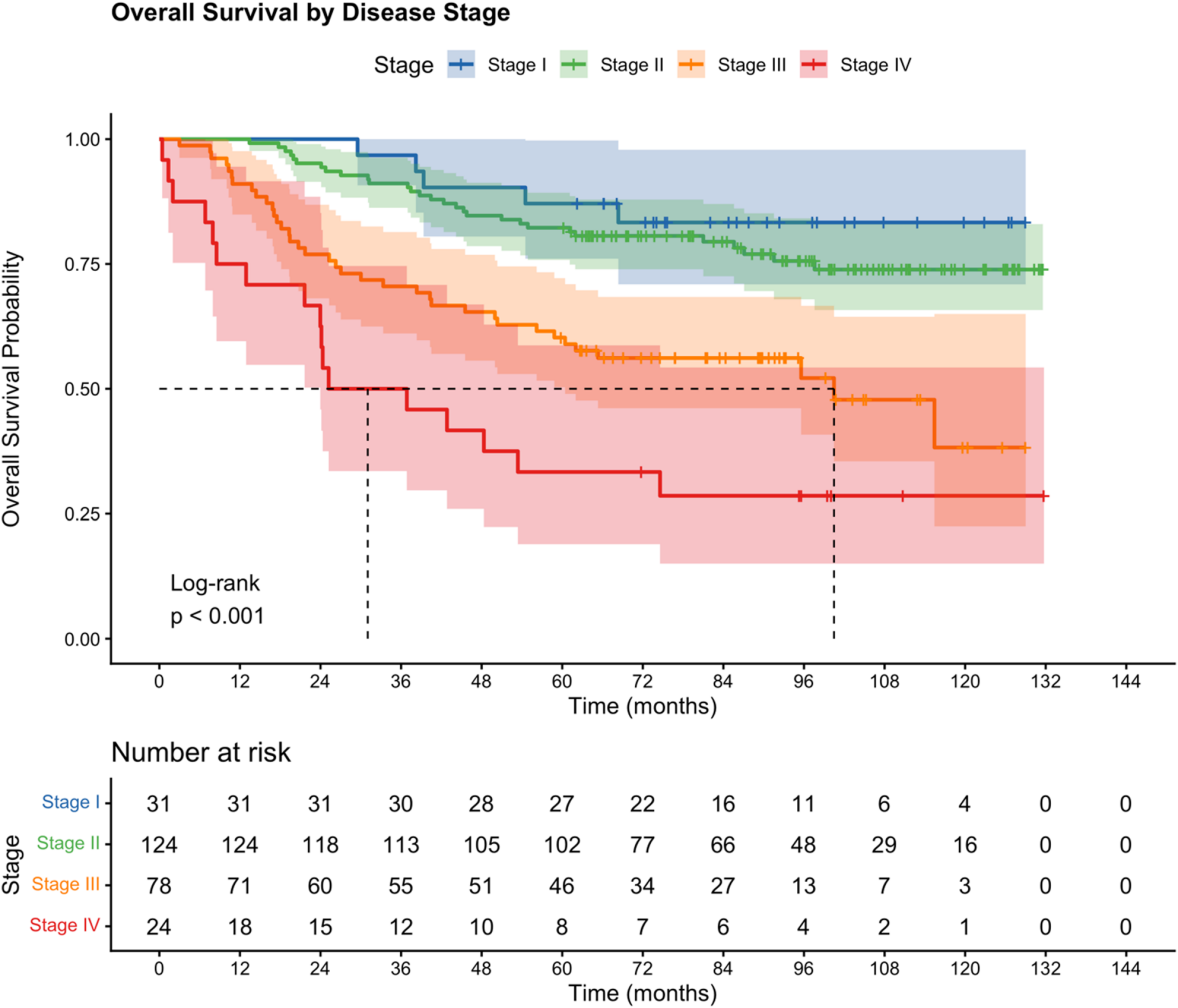
Kaplan–Meier curves for overall survival according to disease stage. Log-rank *p* < 0.001. Numbers at risk are shown below the x-axis. Shaded areas represent 95% confidence intervals. Tick marks indicate censored observations

Survival rates differed markedly by disease stage (log-rank *p* < 0.001). Kaplan–Meier survival curves by disease stage are presented in Fig. 1.

Among Stage I patients, the 3-year and 5-year OS were 96.8% (95% CI: 90.8–100%) and 87.1% (95% CI: 76.1–99.7%) respectively, with median OS not reached. For Stage II patients, 3-year and 5-year OS were 91.1% (95% CI: 86.3–96.3%) and 82.3% (95% CI: 75.8–89.3%) respectively. For Stage III patients, 3-year OS was 70.5% (95% CI: 61.1–81.4%) and 5-year OS was 60.3% (95% CI: 50.3–72.2%), with a median OS of 100.4 months (95% CI: 60.4 months–not reached). Stage IV patients had substantially poorer outcomes, with 3-year OS of 50.0% (95% CI: 33.5–74.6%), 5-year OS of 33.3% (95% CI: 18.9–58.7%), and a median OS of 31.0 months (95% CI: 23.9 months–not reached). Detailed Kaplan–Meier survival estimates at 3 and 5 years by disease stage and molecular subtype are presented in Table 2.

**Table 2:** Univariable Cox proportional hazards regression for overall survival

Characteristic	*N*	HR	95% CI	*p*-value
Age (years)	293	0.99	0.95, 1.02	0.4
BMI (kg/m²)	293	0.98	0.95, 1.03	0.5
Disease stage	257			
Stage I (ref)		—	—	
Stage II		1.49	0.58, 3.86	0.4
Stage III		3.86	1.52, 9.84	0.005*
Stage IV		7.82	2.88, 21.2	< 0.001*
Tumor grade	254			
Grade I (ref)		—	—	
Grade II		3.30	1.31, 8.31	0.011*
Grade III		4.49	1.78, 11.3	0.001*
Molecular subtype	269			
HR+/HER2- (ref)		—	—	
HR+/HER2+		2.31	1.38, 3.88	0.002*
HR-/HER2+		2.65	1.44, 4.90	0.002*
HR-/HER2-		2.25	1.40, 3.64	< 0.001*
Surgery	293			
Yes (ref)		—	—	
No		9.06	5.96, 13.8	< 0.001*
Chemotherapy	292			
No (ref)		—	—	
Yes		1.54	0.94, 2.53	0.085
Hormone therapy	186			
No (ref)		—	—	
Yes		0.27	0.16, 0.45	< 0.001*

*HR *Hazard Ratio,* CI *Confidence Interval,* Ref *reference category

* *p* < 0.05

**Fig. 2:**
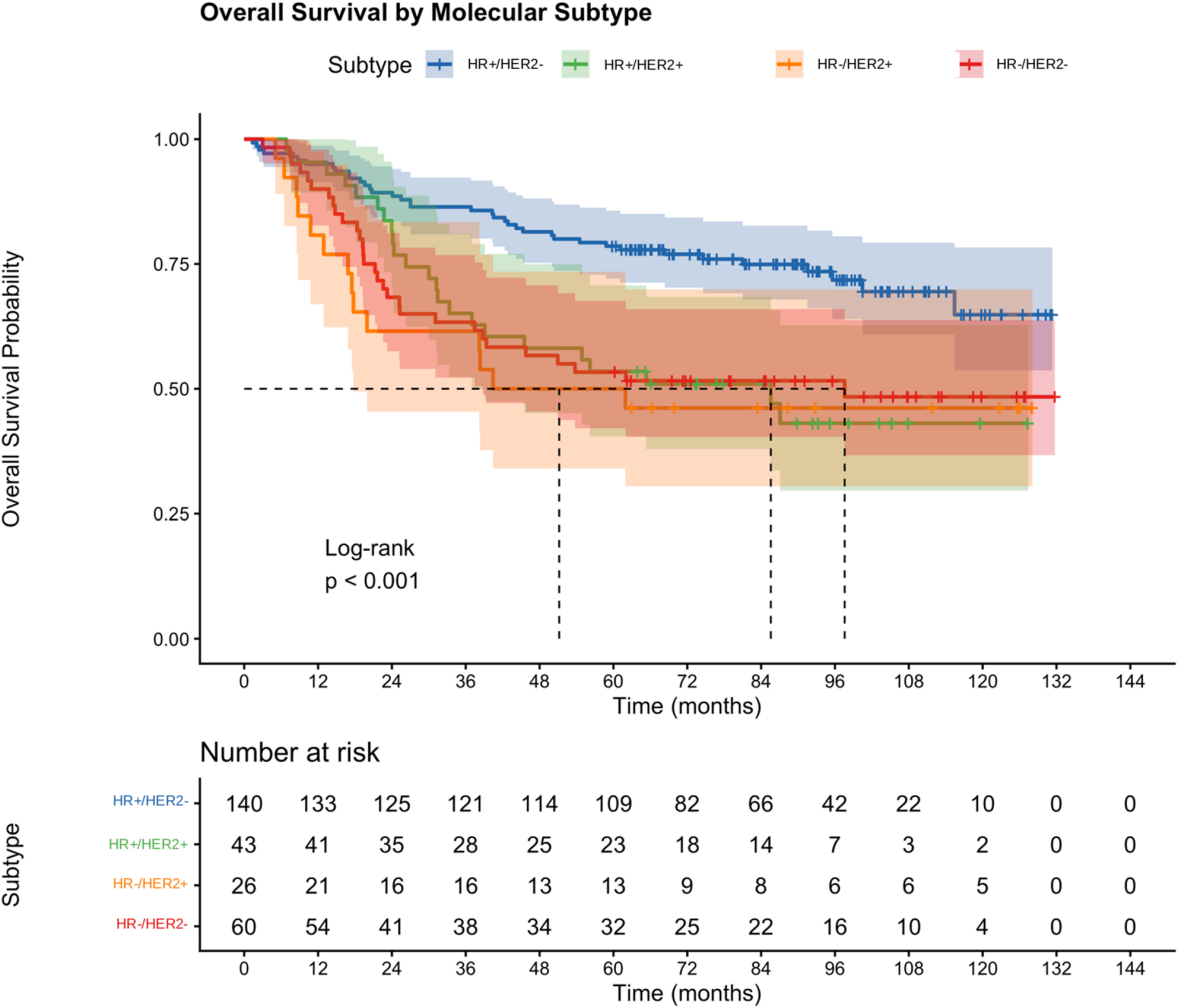
Kaplan–Meier curves for overall survival according to molecular subtype. Log-rank *p* < 0.001. Molecular subtypes were classified using immunohistochemistry surrogate criteria per the St. Gallen 2013 consensus. Numbers at risk are shown below the x-axis. Shaded areas represent 95% confidence intervals. Tick marks indicate censored observations

Survival also differed significantly by molecular subtype (log-rank *p* < 0.001; Fig. 2). HR+/HER2- patients had the most favourable 5-year OS of 78.6% (95% CI: 72.1–85.7%). HR+/HER2+, HR-/HER2+, and HR-/HER2- subtypes demonstrated lower 5-year OS rates of 53.5% (95% CI: 40.5–70.7%), 50.0% (95% CI: 34.0–73.4%), and 53.3% (95% CI: 42.1–67.6%) respectively.

### Disease-free and distant disease-free survival

One hundred patients (34.1%) experienced disease recurrence during follow-up. Recurrence was locoregional only in 40 patients (13.7%), distant in 73 patients (24.9%), and both locoregional and distant in 7 patients (2.4%). The median time to recurrence was 25.5 months.

**Fig. 3:**
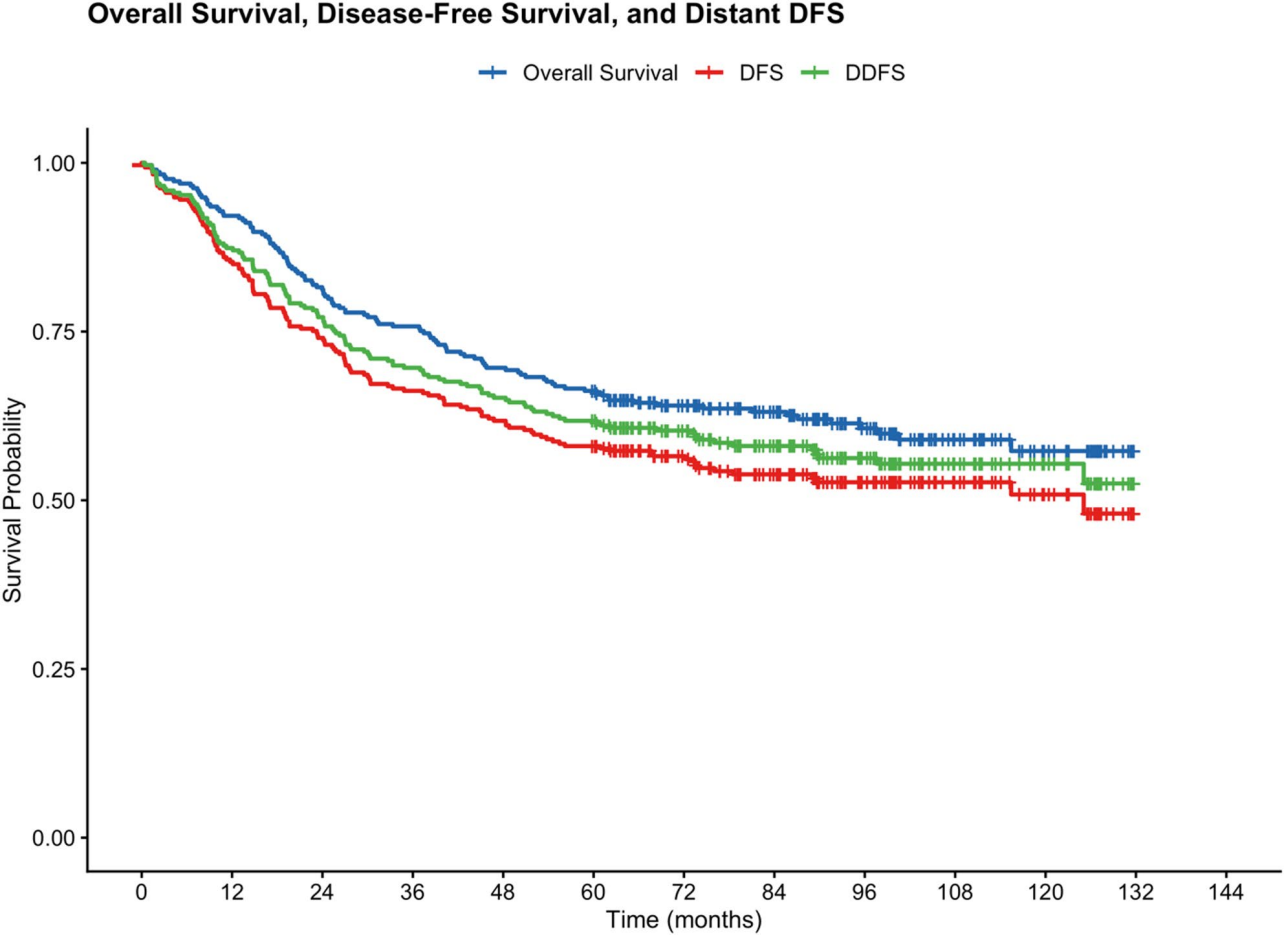
Kaplan–Meier curves for overall survival (OS), disease-free survival (DFS), and distant disease-free survival (DDFS). DFS was defined as time from diagnosis to first recurrence at any site or death from any cause. DDFS was defined as time from diagnosis to first distant recurrence or death from any cause. The narrow gap between OS and DDFS confirms minimal competing non-cancer mortality in this young cohort. Tick marks indicate censored observations

**Fig. 4:**
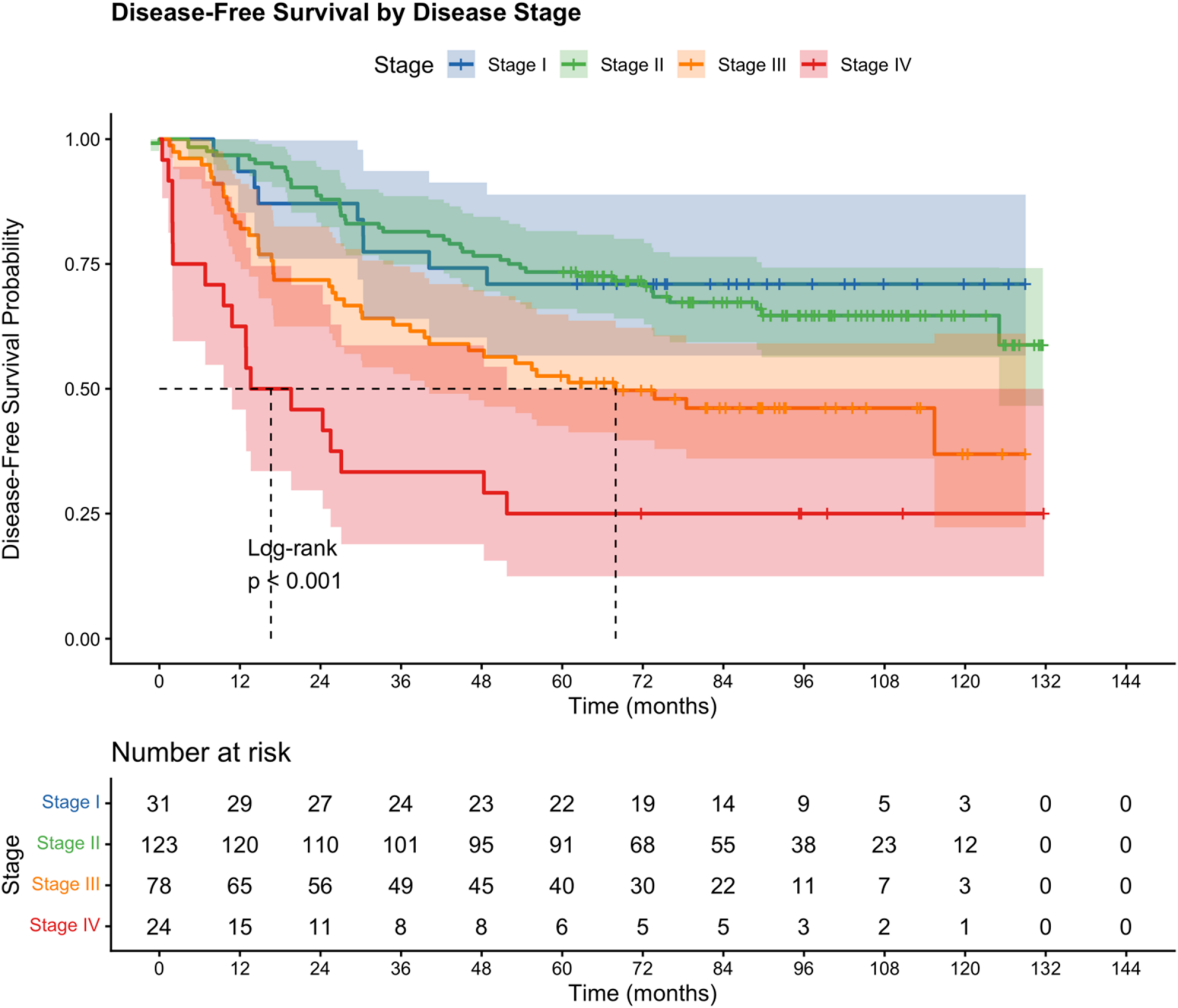
Kaplan–Meier curves for disease-free survival according to disease stage. Log-rank *p* < 0.001. Numbers at risk are shown below the x-axis. Shaded areas represent 95% confidence intervals. Tick marks indicate censored observations

The 3-year and 5-year DFS rates were 66.2% (95% CI: 61.0–71.9%) and 58.0% (95% CI: 52.6–64.0%), respectively, with a median DFS of 125.0 months. The 3-year and 5-year DDFS rates were 69.6% (95% CI: 64.6–75.1%) and 61.8% (95% CI: 56.5–67.6%), respectively, with median DDFS not reached. Five-year DFS by disease stage was: Stage I 71.0% (95% CI: 56.7–88.9%), Stage II 73.4% (95% CI: 66.0–81.6%), Stage III 52.6% (95% CI: 42.6–64.9%), and Stage IV 25.0% (95% CI: 12.5–50.0%). Kaplan–Meier curves for OS, DFS, and DDFS are presented in Fig. 3, and DFS by disease stage in Fig. 4. The narrow gap between OS and DDFS at all timepoints (4.4% points at 5 years) confirms that competing non-cancer mortality was minimal in this young cohort.

### Univariable Cox proportional hazards analysis

Results of the univariable Cox proportional hazards analysis are presented in Table 3. Increasing disease stage was strongly associated with poorer overall survival. Compared with Stage I disease, patients with Stage III (HR = 3.86, 95% CI: 1.52–9.84, *p* = 0.005) and Stage IV disease (HR = 7.82, 95% CI: 2.88–21.2, *p* < 0.001) had significantly increased hazards of death. Tumour grade was significantly associated with survival: compared with Grade I, patients with Grade II (HR = 3.30, 95% CI: 1.31–8.31, *p* = 0.011) and Grade III tumours (HR = 4.49, 95% CI: 1.78–11.3, *p* = 0.001) had substantially higher hazards of death.

**Table 3:** Multivariable Cox proportional hazards regression for overall survival (Multiple Imputation, m = 10**)** *N* = 211 patients (complete for stage, grade, subtype, surgery); hormone therapy imputed using MICE (107 missing, 36.5%). Events per variable ratio = 11.4

Characteristic	aHR	95% CI	*p*-value
Disease stage			
Stage I (ref)	—	—	—
Stage II	1.11	0.33–93.71	0.857
Stage III	2.99	0.92–99.66	0.067
Stage IV	1.63	0.37–97.08	0.509
Tumor grade			
Grade I (ref)	—	—	—
Grade II	2.68	0.77–99.34	0.119
Grade III	2.49	0.68–99.12	0.166
Molecular subtype			
HR+/HER2- (ref)	—	—	—
HR+/HER2+	**2.08**	**1.07–94.07**	**0.032**
HR-/HER2+	1.98	0.83–94.72	0.118
HR-/HER2-	1.57	0.48–95.09	0.423
Surgery			
Yes (ref)	—	—	—
No	**11.60**	**3.19–942.16**	**0.001**
Hormone therapy			
No (ref)	—	—	—
Yes	**0.20**	**0.10–90.42**	**0.001**

Results derived from multiple imputation (m = 10, MICE) pooled using Rubin’s Rules

Reference categories: Stage I, Grade I, HR+/HER2-, Surgery Yes, Hormone therapy No. Bold values indicate *p* < 0.05

*aHR* adjusted Hazard Ratio, *CI* Confidence Interval

Regarding molecular subtype, all non-HR+/HER2- subtypes demonstrated significantly higher hazards of death compared with HR+/HER2-: HR+/HER2+ (HR = 2.31, 95% CI: 1.38–3.88, *p* = 0.002), HR-/HER2+ (HR = 2.65, 95% CI: 1.44–4.90, *p* = 0.002), and HR-/HER2- (HR = 2.25, 95% CI: 1.40–3.64, *p* < 0.001).

Receipt of surgery was strongly associated with overall survival in univariable analysis (HR for no surgery = 9.06, 95% CI: 5.96–13.8, *p* < 0.001); however, this association primarily reflects disease operability rather than an isolated surgical treatment effect, as patients who did not undergo surgery were predominantly those with advanced or palliative-intent disease (see non-surgery group description below). Receipt of hormone therapy was associated with better overall survival (HR = 0.27, 95% CI: 0.16–0.45, *p* < 0.001). Chemotherapy was not significantly associated with overall survival in univariable analysis (HR = 1.54, 95% CI: 0.94–2.53, *p* = 0.085), likely reflecting confounding by indication, as 86.0% of patients received chemotherapy with the small non-chemotherapy group representing a heterogeneous mix of early-stage and advanced palliative patients. Adjuvant radiotherapy could not be reliably estimated due to incomplete documentation, as described in the Methods section.

### Non-surgery group

Of the 37 patients (12.6%) who did not receive surgery, 25 (67.6%) had missing disease stage, 26 (70.3%) received palliative chemotherapy, and 34 (91.9%) died during follow-up, with a median OS of only 16.4 months. This profile confirms that the non-surgery group represented a systematically advanced, predominantly inoperable population rather than a clinically comparable group of operable patients who declined surgery. Receipt of surgery is therefore best interpreted as a marker of disease operability rather than an independent causal treatment effect.

### Multivariable Cox proportional hazards analysis

Results of the revised multivariable Cox proportional hazards analysis using multiple imputation (m = 10 imputed datasets) are presented in Table 4. The model included five covariates: disease stage, tumour grade, molecular subtype, surgery, and hormone therapy, with an events-per-variable ratio of 11.4.

**Table 4:** Kaplan–Meier overall survival estimates at 3 and 5 years by disease stage and molecular subtype *N* = 293 patients. Median follow-up 93.3 months (95% CI: 89.8–98.2 months)

Group	*n*	3-year OS (95% CI)	5-year OS (95% CI)	Median OS
Overall	293	75.8% (71.0-80.8%)	66.2% (61.0-71.9%)	Not reached
By stage				
Stage I	31	96.8% (90.8–100%)	87.1% (76.1–99.7%)	Not reached
Stage II	124	91.1% (86.3–96.3%)	82.3% (75.8–89.3%)	Not reached
Stage III	78	70.5% (61.1–81.4%)	60.3% (50.3–72.2%)	100.4 months (60.4-NR)
Stage IV	24	50.0% (33.5–74.6%)	33.3% (18.9–58.7%)	31.0 months (23.9-NR)
By subtype				
HR+/HER2-	140	—	78.6% (72.1–85.7%)	Not reached
HR+/HER2+	43	—	53.5% (40.5–70.7%)	Not reached
HR-/HER2+	26	—	50.0% (34.0-73.4%)	Not reached
HR-/HER2-	60	—	53.3% (42.1–67.6%)	Not reached

Kaplan–Meier estimates with Greenwood standard errors. Subtype 3-year OS not calculated due to incomplete subtype data collection at earlier timepoints

*OS* overall survival, *NR* not reached, *CI* confidence interval

— = not calculated

In multivariable analysis, HR+/HER2 + subtype was independently associated with poorer overall survival compared with HR+/HER2- (aHR = 2.08, 95% CI: 1.07–4.07, *p* = 0.032). Hormone therapy receipt was independently associated with better overall survival (aHR = 0.20, 95% CI: 0.10–0.42, *p* < 0.001). Receipt of surgery remained strongly associated with overall survival (aHR for no surgery = 11.60, 95% CI: 3.19–42.16, *p* < 0.001), noting that this reflects disease operability as described above. Disease stage and tumour grade did not reach statistical significance in the multivariable model, likely reflecting the competing effects of molecular subtype and treatment variables in adjustment, together with the relatively modest sample size. HR-/HER2 + subtype and HR-/HER2- did not reach statistical significance after multivariable adjustment (*p* = 0.118 and *p* = 0.423 respectively), likely due to limited statistical power in these smaller subgroups.

The proportional hazards assumption was confirmed for all covariates (global Schoenfeld test: χ²=6.50, df = 9, *p* = 0.69). Sensitivity analyses — including complete case analysis (Supplementary Table S1), Stage I–III restricted analysis (Supplementary Table S2), and MNAR delta adjustment — produced results consistent with the primary analysis. In the Stage I–III restricted sensitivity analysis (*n* = 194, events = 59), HR+/HER2 + remained independently associated with poorer survival (aHR = 2.21, 95% CI: 1.04–4.71, *p* = 0.039) and hormone therapy remained strongly protective (aHR = 0.16, 95% CI: 0.07–0.38, *p* < 0.001). Notably, surgery was inestimable in Stage I–III patients due to near-complete surgery receipt (> 98%), directly confirming its role as an operability surrogate rather than an independent prognostic variable.

## Discussion

This retrospective cohort study provides systematically ascertained, civil registry-linked survival benchmarks for young women (≤ 45 years) with breast cancer at a major provincial referral hospital in Southern Thailand. The overall 5-year overall survival of 66.2% and the marked stage-specific gradient — from 87.1% at Stage I to 33.3% at Stage IV — contextualise the real-world outcomes achievable in this setting. Beyond confirming the expected prognostic roles of disease stage and tumour grade, our revised multivariable analysis identifies HR+/HER2 + molecular subtype and hormone therapy receipt as independent prognostic factors, findings with direct clinical implications for treatment prioritisation in a resource-limited regional setting.

### Disease stage and survival outcomes

Advanced disease stage emerged as the dominant prognostic factor in our cohort in univariable analysis, consistent with extensive evidence from Asian and international populations [2, 3]. The stage-specific 5-year OS rates we report — 87.1% (Stage I), 82.3% (Stage II), 60.3% (Stage III), and 33.3% (Stage IV) — are lower than those typically reported in high-income country series, where 5-year OS for Stage I and II breast cancer commonly exceeds 90–95% [19]. This survival gap likely reflects a combination of factors specific to our provincial Southeast Asian setting: limited access to HER2-targeted therapy, lower endocrine therapy completion rates, and the younger age biology of this cohort with its associated higher prevalence of aggressive subtypes.

The relatively high proportion of Stage III disease in our cohort (26.6%) is consistent with patterns of delayed presentation documented in Southeast Asian populations and may partly explain the observed survival disadvantage compared with Western benchmarks [20]. Advanced stage at diagnosis therefore represents both the primary determinant of survival outcomes in our cohort and the primary target for public health intervention in this setting.

### Tumour grade

Higher tumour grade was independently associated with poorer survival in univariable analysis and showed a consistent trend in the multivariable model, consistent with published data from comparable Asian hospital-based cohorts [21]. In young women, the higher prevalence of Grade III tumours compounds the adverse effects of advanced stage and aggressive molecular subtypes. The biological basis relates to greater tumour proliferative activity, genomic instability, and metastatic potential, all of which are more prevalent in younger patients across Asian populations [21].

### Molecular subtypes and biomarkers

The molecular subtype distribution in our cohort — HR+/HER2- 52.0%, HR-/HER2- 22.3%, HR+/HER2 + 16.0%, HR-/HER2 + 9.7% — reflects a higher proportion of non-luminal subtypes compared with Western populations. This pattern is consistent with published data from comparable Southeast Asian and East Asian cohorts, where younger women demonstrate a higher prevalence of HR-/HER2- and HER2-positive disease, and aligns with the known biological shift toward more aggressive subtypes in young Asian women [22].

In our revised multivariable analysis using multiple imputation, HR+/HER2 + subtype was the only molecular subtype independently associated with poorer overall survival compared with HR+/HER2- (aHR = 2.08, 95% CI: 1.07–4.07, *p* = 0.032), a finding confirmed in the Stage I–III restricted sensitivity analysis (aHR = 2.21, 95% CI: 1.04–4.71, *p* = 0.039). This finding has direct clinical relevance in our setting: despite a HER2-positive rate of approximately 26% among classifiable patients, only 4.1% received trastuzumab during the study period due to severe insurance reimbursement restrictions. The independent adverse prognosis of HR+/HER2 + subtype in our cohort may therefore partly reflect the absence of adequate HER2-targeted therapy rather than an intrinsic biological disadvantage over and above what is addressable with treatment. Future studies should examine whether expanded trastuzumab access narrows this survival gap.

HR-/HER2 + and HR-/HER2- subtypes did not reach statistical significance in multivariable analysis, likely reflecting limited statistical power in these smaller subgroups (*n* = 26 and *n* = 60 respectively) rather than an absence of biological effect. The 5-year OS rates for HR-/HER2+ (50.0%) and HR-/HER2- (53.3%) were substantially lower than HR+/HER2- (78.6%), consistent with their known adverse prognosis.

The attenuation of individual ER and PR associations in multivariable analysis — observed in our original model and consistent with published literature — reflects the close correlations between receptor status, tumour grade, stage, and treatment allocation [23]. Replacing separate receptor variables with the composite molecular subtype in our revised model resolves this collinearity and provides more clinically interpretable estimates.

### Treatment effects and confounding

Hormone therapy receipt was independently and strongly associated with better overall survival in both the primary (aHR = 0.20, 95% CI: 0.10–0.42, *p* < 0.001) and Stage I–III sensitivity analyses (aHR = 0.16, *p* < 0.001), with results completely robust to MNAR sensitivity analysis across all delta scenarios tested. However, as with all treatment variables in this retrospective observational cohort, this association should be interpreted as a prognostic marker subject to confounding by indication rather than a direct causal treatment effect estimate. Patients who received hormone therapy were by definition hormone receptor-positive, had survived long enough to initiate treatment, and may have been more adherent to care overall — all factors independently associated with better outcomes.

The observed hormone therapy treatment gap is clinically important: despite the strong protective association, only 51.6% of eligible patients received hormone therapy. Young women are disproportionately affected by endocrine therapy side effects including premature menopause, bone density loss, and fertility concerns, and are at higher risk of premature discontinuation. Our data suggest that addressing adherence barriers and improving access to endocrine therapy in this young population represents the most actionable intervention for improving survival outcomes at our institution.

Receipt of surgery was strongly associated with overall survival (aHR for no surgery = 11.60, *p* < 0.001); however, this association should not be interpreted as a causal treatment effect. As detailed in the Results, the 37 patients who did not receive surgery were predominantly those with palliative-intent disease — 67.6% had missing stage data, 70.3% received palliative chemotherapy, and 91.9% died during follow-up, with a median OS of only 16.4 months. Surgery in this observational cohort functions as a surrogate for disease operability and fitness for curative-intent treatment rather than an independent prognostic variable. This interpretation was directly confirmed by the Stage I–III sensitivity analysis, in which surgery was inestimable due to near-universal receipt (> 98%) within the operable population.

Chemotherapy was not significantly associated with survival in univariable analysis (HR = 1.54, *p* = 0.085) and was excluded from the multivariable model. This likely reflects confounding by indication: the 86.0% who received chemotherapy included a higher proportion of advanced-stage patients with inherently worse prognosis, attenuating the observable benefit. Detailed assessment of chemotherapy efficacy requires randomised or propensity-matched data not available in this retrospective cohort.

### Regional and real-world context

Our findings provide real-world survival data from a provincial referral hospital in Southern Thailand — a setting and population underrepresented in the international breast cancer literature, where most published data derive from national registries, major academic centres in capital cities, or high-income country cohorts. The use of national civil registry linkage for mortality ascertainment substantially reduces the informative censoring that commonly affects retrospective hospital-based survival studies, where patients lost to follow-up may have died at other facilities.

Asian women, including Thai women, are known to develop breast cancer at a younger age than Western populations, with peak incidence occurring approximately one to two decades earlier [24, 25]. Our cohort, with a median age of 40 years, is consistent with this established epidemiological pattern. The molecular subtype distribution — with higher proportions of HR-/HER2- and HR+/HER2 + than typical Western series — aligns with published data from comparable Asian hospital-based cohorts and is likely a contributing factor to the observed survival gap relative to high-income country benchmarks [22, 25]. The survival disadvantage in our cohort therefore reflects a combination of tumour biology, late-stage presentation, and treatment access gaps rather than a single addressable cause.

The relatively lower 5-year OS rates at Stage I (87.1%) and Stage II (82.3%) compared with high-income country benchmarks suggest that improving treatment access — particularly ensuring adequate HER2-targeted therapy and endocrine therapy completion — may offer greater population-level benefit than earlier detection alone in this resource-limited setting. This conclusion is reinforced by the finding that hormone therapy receipt was the most consistent independent prognostic factor across all sensitivity analyses.

### Early detection and breast health awareness

Given the high proportion of Stage III disease at presentation (26.6%) and the significant mortality burden documented in this cohort, targeted strategies to promote earlier diagnosis and breast health awareness are warranted. In resource-limited settings such as Southern Thailand, where population-wide mammographic screening for younger women is not feasible, a risk-stratified approach to early detection may offer the most practical benefit. Breast self-examination education and promotion of clinical breast examinations at routine primary care visits represent low-cost, accessible interventions that may reduce diagnostic delays in younger women who fall outside standard mammographic screening age thresholds. Healthcare providers in primary care and community health settings should be equipped to recognise early warning signs in women aged 20–45 years, who are frequently not prioritised in conventional screening programmes. Community-based breast health outreach — particularly in the geographically dispersed and ethnically diverse communities served by our institution, including both Buddhist Thai and Muslim Thai populations — may further support earlier presentation. Beyond detection, addressing the identified hormone therapy treatment gap, in which only 51.6% of eligible patients received endocrine therapy, requires structured adherence support and patient education to mitigate the specific barriers faced by young women, including concerns about premature menopause, fertility implications, and long-term side effects. Future research should evaluate the feasibility, acceptability, and cost-effectiveness of culturally appropriate early detection models tailored to the epidemiological and resource context of provincial Southeast Asian settings.

### Limitations

Several limitations warrant consideration. First, as a retrospective observational study, findings are subject to residual confounding and selection bias, particularly regarding treatment variables. Causal inferences about treatment efficacy cannot be drawn from these data. Second, the study was restricted to women aged ≤ 45 years, which differs from the ≤ 49 years bracket used in some international series, limiting direct numerical comparison. Patients aged 46–49 years were not enrolled as the age cutoff was applied at recruitment. Third, cause-of-death data were not consistently available, precluding breast cancer-specific survival analysis; however, the narrow gap between OS and DDFS (4.4% points at 5 years) confirms that competing non-cancer mortality was minimal in this young cohort, and OS is considered a close approximation of breast cancer-specific survival. Fourth, trastuzumab access was severely restricted during the study period (4.1% of patients), limiting assessment of HER2-targeted therapy impact and potentially contributing to the adverse prognosis of HER2-positive subtypes. Fifth, family history, BRCA mutation status, and genetic risk assessment data were not available, as genetic counselling and testing were not routinely available at our institution during the study period. Sixth, reasons for non-receipt of hormone therapy were not systematically recorded, precluding analysis of treatment refusal versus access barriers. Seventh, missing data — particularly for hormone therapy (36.5%) and disease stage (12.3%) — were addressed using multiple imputation under the MAR assumption; MNAR cannot be entirely excluded, though delta-adjusted sensitivity analysis confirmed robustness of the hormone therapy finding to potential MNAR departures. Finally, as a single-centre study, findings may not be fully generalisable to other provincial Thai hospitals with different patient populations or resource availability.

## Conclusion

This study provides systematically ascertained, registry-linked survival benchmarks for young women (≤ 45 years) with breast cancer at a major provincial referral hospital in Southern Thailand. The 5-year overall survival of 66.2% and the marked stage-specific survival gradient confirm the prognostic dominance of disease stage in this population. In multivariable analysis using multiple imputation, HR+/HER2 + molecular subtype was independently associated with poorer survival compared with HR+/HER2-, and hormone therapy receipt was independently associated with better overall survival — findings that were robust across all sensitivity analyses including MNAR adjustment. Receipt of surgery was strongly associated with survival but largely reflected disease operability rather than an independent treatment effect.

The relatively lower stage-specific survival rates compared with high-income country benchmarks highlight the need for improved access to HER2-targeted therapy and endocrine therapy in this resource-limited setting. The hormone therapy treatment gap identified in our cohort — with only 51.6% of eligible patients receiving endocrine therapy despite its strong independent protective association — represents the most actionable target for improving survival outcomes. These findings provide a foundation for prognostic counselling, treatment planning, and healthcare resource allocation at comparable provincial Southeast Asian centres, and support the need for future prospective studies with complete treatment data, longer follow-up, and systematic genetic risk assessment in young Thai women with breast cancer.

## Supplementary Information

Supplementary Material 1.

Supplementary Material 2.

Supplementary Material 3.

Supplementary Material 4.

## Data Availability

The datasets generated and/or analyzed during the current study are not publicly available due to institutional and ethical restrictions related to patient confidentiality but are available from the corresponding author on reasonable request and with permission from Hat Yai Hospital.
