# Supplementary material for: Overall survival and prognostic factors in young women with breast cancer: a retrospective cohort study from Southern Thailand
Source: World J Surg Oncol. 2026 Apr 15;24:229. doi: 10.1186/s12957-026-04349-9 (PMC13195995; doi:10.1186/s12957-026-04349-9)
Supplement: Supplementary file 1 — Supplementary Material 1. [file 12957_2026_4349_MOESM1_ESM.docx]

**Supplementary Table S1.** Complete case sensitivity analysis: multivariable Cox proportional hazards regression for overall survival.

*Complete case analysis restricted to patients with no missing values across all five model covariates (disease stage, tumour grade, molecular subtype, surgery, and hormone therapy). N=148 patients; 45 events (deaths); events-per-variable ratio=4.5.*

| **Characteristic** | **aHR** | **95% CI** | **p-value** |
| --- | --- | --- | --- |
| **Disease stage** |  |  |  |
| Stage I (ref) | — | — |  |
| Stage II | 1.18 | 0.32–4.38 | 0.799 |
| Stage III | 3.24 | 0.97–10.87 | 0.057 |
| Stage IV | 1.91 | 0.45–8.04 | 0.378 |
| **Tumour grade** |  |  |  |
| Grade I (ref) | — | — |  |
| Grade II | 2.45 | 0.62–9.65 | 0.200 |
| Grade III | 2.88 | 0.72–11.55 | 0.137 |
| **Molecular subtype** |  |  |  |
| Luminal A (ref) | — | — |  |
| Luminal B (HER2+) | **2.14** | **1.03–4.47** | **0.043** |
| HER2-enriched | *Inestimable* |  | *N/A* |
| TNBC | *Inestimable* |  | *N/A* |
| **Surgery** |  |  |  |
| Yes (ref) | — | — |  |
| No | **10.24** | **2.18–48.11** | **0.003** |
| **Hormone therapy** |  |  |  |
| No (ref) | — | — |  |
| Yes | **0.22** | **0.10–0.48** | **<0.001** |

*aHR = adjusted Hazard Ratio; CI = Confidence Interval; N/A = not applicable. Bold p-values indicate p<0.05. HER2-enriched (n=0 after listwise deletion) and TNBC (n=1 after listwise deletion) subtypes were inestimable due to near-complete exclusion of receptor-negative patients, who disproportionately had missing hormone therapy data. This confirms the limitation of complete case analysis for this dataset and supports multiple imputation as the primary analytical approach. Key findings for Luminal B (HER2+) subtype (aHR=2.14, p=0.043), surgery (aHR for no surgery=10.24, p=0.003), and hormone therapy (aHR=0.22, p<0.001) are consistent with the primary multiple imputation analysis.*
