# Supplementary material for: Overall survival and prognostic factors in young women with breast cancer: a retrospective cohort study from Southern Thailand
Source: World J Surg Oncol. 2026 Apr 15;24:229. doi: 10.1186/s12957-026-04349-9 (PMC13195995; doi:10.1186/s12957-026-04349-9)
Supplement: Supplementary file 2 — Supplementary Material 2. [file 12957_2026_4349_MOESM2_ESM.docx]

**Supplementary Table S2.** Stage I–III restricted sensitivity analysis: multivariable Cox proportional hazards regression for overall survival, excluding Stage IV (metastatic) patients.

*Analysis restricted to patients with Stage I–III disease and complete data for stage, tumour grade, molecular subtype, and surgery. Hormone therapy was imputed using multiple imputation by chained equations (MICE, m=10). N=194 patients; 59 events (deaths); events-per-variable ratio=5.9. This analysis was conducted to avoid conflating the prognostic role of surgical treatment with the inherent survival disadvantage of Stage IV metastatic disease.*

| **Characteristic** | **aHR** | **95% CI** | **p-value** |
| --- | --- | --- | --- |
| **Disease stage** |  |  |  |
| Stage I (ref) | — | — |  |
| Stage II | 1.20 | 0.33–4.42 | 0.773 |
| Stage III | 3.16 | 0.90–11.00 | 0.070 |
| **Tumour grade** |  |  |  |
| Grade I (ref) | — | — |  |
| Grade II | 2.30 | 0.65–8.08 | 0.190 |
| Grade III | 1.71 | 0.45–6.44 | 0.421 |
| **Molecular subtype** |  |  |  |
| Luminal A (ref) | — | — |  |
| Luminal B (HER2+) | **2.21** | **1.04–4.71** | **0.039** |
| HER2-enriched | 1.72 | 0.52–5.74 | 0.361 |
| TNBC | 2.57 | 0.69–9.61 | 0.144 |
| **Surgery** |  |  |  |
| Yes (ref) | — | — |  |
| No | *Inestimable — see footnote* |  | *N/A* |
| **Hormone therapy** |  |  |  |
| No (ref) | — | — |  |
| Yes | **0.16** | **0.07–0.38** | **<0.001** |

*aHR = adjusted Hazard Ratio; CI = Confidence Interval; N/A = not applicable. Results pooled from 10 imputed datasets using Rubin’s Rules. Bold p-values indicate p<0.05. Surgery was inestimable due to near-universal receipt among Stage I–III patients (>98% underwent surgery), directly confirming that surgery functions as a surrogate for disease operability rather than an independent prognostic variable in this observational cohort. Luminal B (HER2+) subtype (aHR=2.21, 95% CI: 1.04–4.71, p=0.039) and hormone therapy (aHR=0.16, 95% CI: 0.07–0.38, p<0.001) remained independently associated with overall survival, consistent with the primary multiple imputation analysis (Luminal B aHR=2.08, p=0.032; hormone therapy aHR=0.20, p<0.001).*
