# Supplementary material for: Overall survival and prognostic factors in young women with breast cancer: a retrospective cohort study from Southern Thailand
Source: World J Surg Oncol. 2026 Apr 15;24:229. doi: 10.1186/s12957-026-04349-9 (PMC13195995; doi:10.1186/s12957-026-04349-9)
Supplement: Supplementary file 3 — Supplementary Material 3. [file 12957_2026_4349_MOESM3_ESM.pptx]

## Slide 1
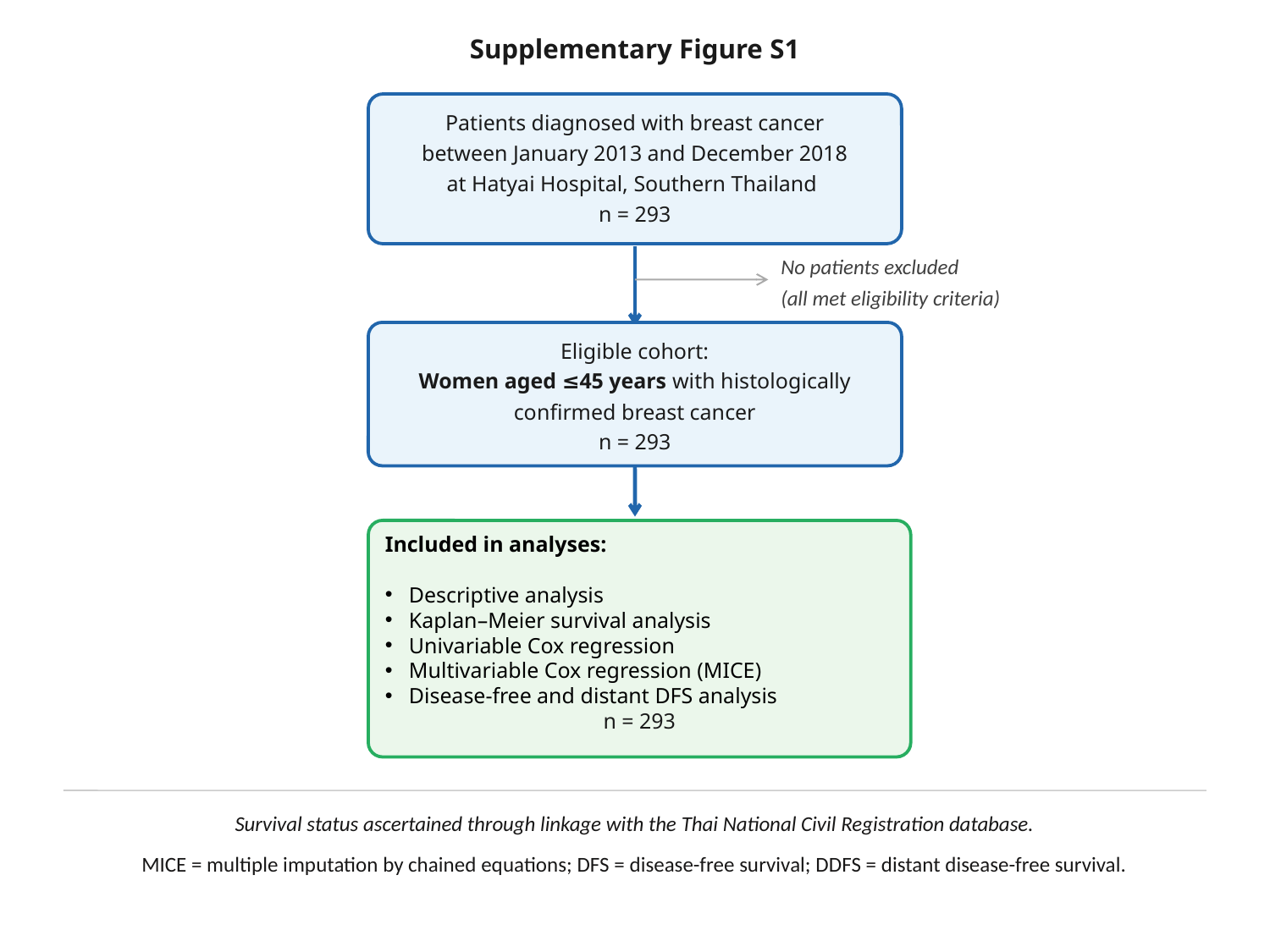

Supplementary Figure S1
Patients diagnosed with breast cancer
between January 2013 and December 2018
at Hatyai Hospital, Southern Thailand
n = 293
No patients excluded
(all met eligibility criteria)
Eligible cohort:
Women aged ≤45 years with histologically
confirmed breast cancer
n = 293
Included in analyses:
Descriptive analysis
Kaplan–Meier survival analysis
Univariable Cox regression
Multivariable Cox regression (MICE)
Disease-free and distant DFS analysis
n = 293
Survival status ascertained through linkage with the Thai National Civil Registration database.
MICE = multiple imputation by chained equations; DFS = disease-free survival; DDFS = distant disease-free survival.
