# Supplementary figures and images for: Overall survival and prognostic factors in young women with breast cancer: a retrospective cohort study from Southern Thailand
Source: World J Surg Oncol. 2026 Apr 15;24:229. doi: 10.1186/s12957-026-04349-9 (PMC13195995; doi:10.1186/s12957-026-04349-9)

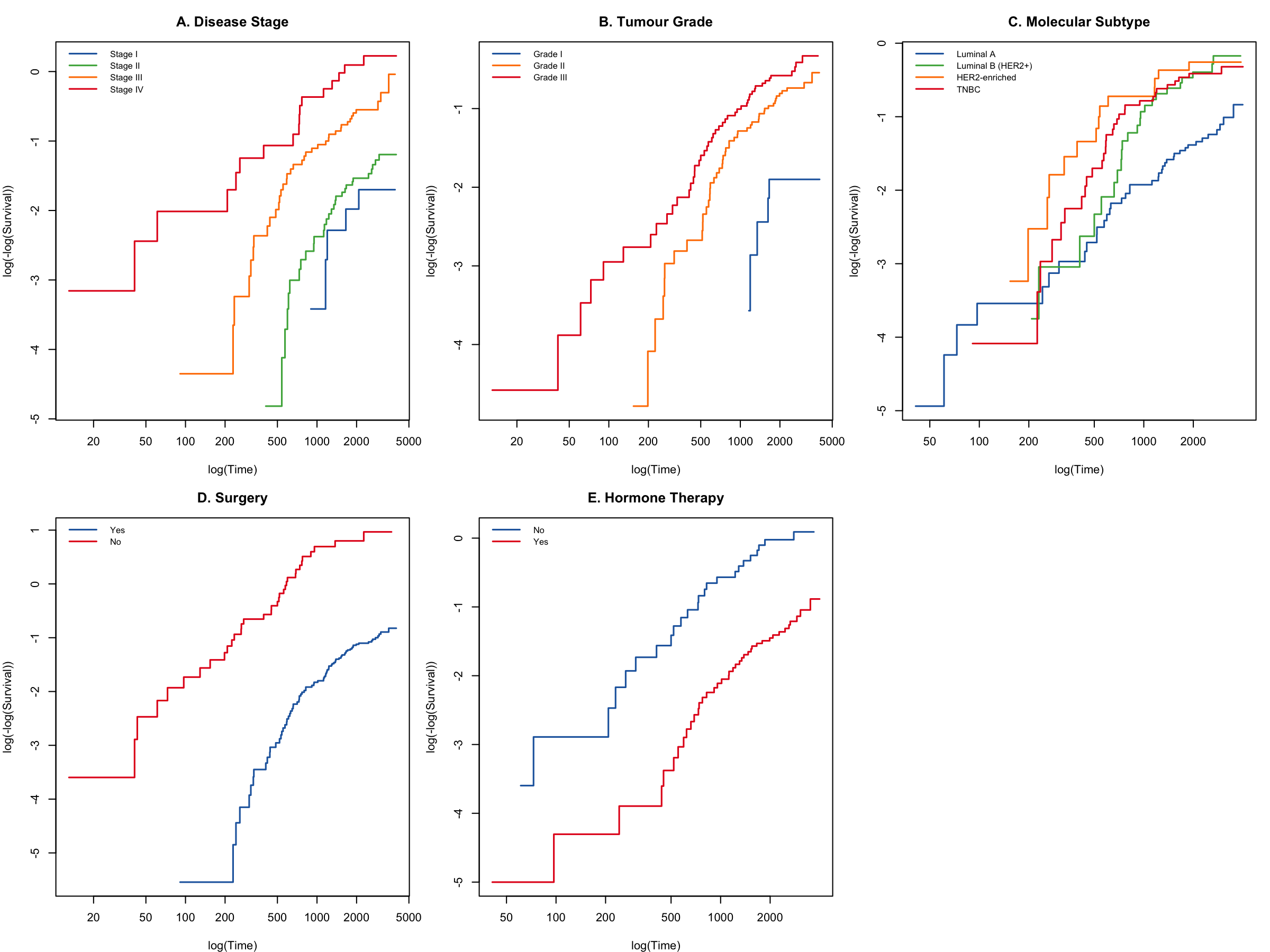

Supplement: Supplementary file 4 — Supplementary Material 4. [file 12957_2026_4349_MOESM4_ESM.png]
